# Ordered Mesoporous Nanomaterials

**DOI:** 10.3390/nano4040902

**Published:** 2014-12-03

**Authors:** Eva Pellicer, Jordi Sort

**Affiliations:** 1 Departament de Física, Universitat Autònoma de Barcelona, E-08193 Bellaterra, Spain; 2 Institució Catalana de Recerca i Estudis Avançats (ICREA) and Departament de Física, Universitat Autònoma de Barcelona, E-08193 Bellaterra, Spain

The Special Issue of *Nanomaterials* “Ordered Mesoporous Nanomaterials” covers novel synthetic aspects of mesoporous materials and explores their use in diverse areas like drug delivery, photocatalysis, filtration or electrocatalysis. The range of materials tackled includes metals and alloys, aluminosilicates, silica, alumina and transition metal oxides. The variety of materials, synthetic approaches and applications examined is vivid proof of the interest that mesoporous materials spark among researchers world-wide.

In this issue, special attention is paid to mesoporous metallic materials. For example, Dumée and co-workers [1] investigate the effects of initial microstructure of Cu_85_Zn_15_, Cu_70_Zn_30_ and Au_50_Ag_50_ foils and de-alloying kinetics on the morphology of the chemically de-alloyed Cu and Au porous thin films. A mechanism for pore formation, which takes into account initial foil thickness, phase distribution, and grain boundaries, is proposed. In another contribution from the same group, the synthesis of porous Ag and Cu thin films from silica nanoparticles by a novel cold-sintering method followed by selective etching of the silica template is presented [2]. Gas permeation studies were conducted to prove the formation of pores throughout the whole body structure. Pore size distribution, porosity and specific surface area are found to be closely related to the silica content before the etching step. Serrà *et al.* [3] report on the fabrication of mesoporous CoPt nanowires by electrodeposition in polycarbonate membranes with nominal pore size of 200 nm. An unconventional ionic liquid-in-water microemulsion is used as the electrolyte. Since cobalt and platinum salts only dissolve in the aqueous component of the electrolyte, the alloy is deposited inside the channels of the membrane from the aqueous phase, whereas nanometer-sized pores develop from the small ionic liquid droplets. The mesoporous CoPt nanowires exhibit higher activity toward electrocatalytic oxidation of methanol than fully-dense nanowires of the same composition.

Several synthetic aspects and properties of ordered mesoporous silica, aluminosilicate and alumina materials are also examined in this issue. Martin and co-workers [4] report on the synthesis of ordered mesoporous SBA-15 analogs with different Si/Al ratios assembled from nanosized ZSM-5 precursors, under high temperature and acidic conditions. These analogs (with pore size *ca*. 9 nm) show an increased density of Brønsted acid sites compared to conventional Al-SBA-15 material. This may open new possibilities for the catalytic conversion of bulky molecules. On the other hand, Canning *et al.* [5] investigate the percolation diffusion properties of 11.5 cm long, self-assembled silica microfibers by optical means. The authors demonstrate that Rhodamine B can penetrate and diffuse through the microfibers, whereas other larger molecules, such as zinc tetraphenylporphyrin, cannot, which therefore proves the great potential of these microfibers as molecular sieves for the separation of biological molecules. Likewise, Romero *et al.* [6] determine relevant electrolyte diffusive transport parameters through self-ordered alumina membranes synthesized by two-step anodization method. Membrane potential in NaCl solutions is measured in a series of alumina membranes with similar pore sizes (23–24 nm in diameter) but different porosity, under both stagnant and stirring conditions. Deep analysis of the results obtained reveal that Donnan exclusion of co-ions at the solution/membrane interface seem to exert a certain control on the diffusive transport of ions through the membranes with lower porosity. This can be reduced by coating the surface of the membranes with SiO_2_. One of the most familiar applications of mesoporous silica, namely as drug delivery platform, is explored by Garcia-Bennet and co-workers [7]. The encapsulation of PA-824, a bactericidal agent against tuberculosis, is studied and the antibacterial activity in tuberculosis-infected macrophages is further analyzed. According to the authors, PA-824 can be successfully loaded into mesoporous AMS-6 nanoparticles and the solubility and rate of PA-824 in *in vitro* kinetic release studies is enhanced compared to the poorly-soluble free PA-824 drug.

Finally, the synthesis of mesoporous titania by evaporation-induced self-assembly method is reported by Masolo *et al.* [8]. The impact of titania precursor, calcination rate and ligand addition on the morphology, texture, and phase percentages (*i.e.*, anatase, rutile and brookite contents) of the resulting powders is systematically investigated. Mesoporous titania with narrower pore sizes and higher anatase contents prepared from acetylacetonate precursor displays remarkable photoelectrochemical activity, higher than commercial P25.

In summary, this Special Issue provides a short collection of the latest developments on mesoporous materials, both from synthetic and applied viewpoints. We hope that the readers will enjoy the articles this Special Issue brings together and will stimulate further research in the field.
